# A differential expression of an identical mutation in *CYP17A1* gene in two infertility patients: a case report

**DOI:** 10.1186/s13256-024-04654-5

**Published:** 2024-07-23

**Authors:** Elay Rabinovich, Anat Hershko-Klement, Yaakov Bentov

**Affiliations:** 1 Department of Military Medicine and “Tzameret”, Faculty of Medicine, Hebrew University of Jerusalem, Jerusalem, Israel; 2 Medical Corps, Israel Defence Forces, Jerusalem, Israel; 3 Faculty of Medicine, Hebrew University of Jerusalem, Jerusalem, Israel; 4 Division of Obstetrics and Gynecology, Hadassah-Hebrew University Medical Center, Jerusalem, Israel; 5 Department of Obstetrics and Gynecology, Hadassah Mount Scopus-Hebrew University Medical Center, 91240 Jerusalem, Israel

**Keywords:** Congenital adrenal hyperplasia (CAH), 17α-Hydroxylase, Deficiency, Infertility, Steroidogenesis, Case report

## Abstract

**Background:**

17-Hydroxylase deficiency is the rarest form of congenital adrenal hyperplasia, a disorder that affects steroidogenesis, causing abnormal hormone levels. Studies have shown a clear association between 17-hydroxylase deficiency and primary infertility, but a definite protocol to treat the disorder has not been determined yet.

**Case presentation:**

Case I presents a 24-year-old Caucasian Israeli-Arab female who experienced 6 years of infertility. Before her initial visit to our clinic, she underwent three laparoscopic ovarian cystectomies, had an unsuccessful *in vitro* fertilization cycle, and was treated with combined oral contraceptives. Her hormonal profile was tested, and the results led to genetic counseling and the diagnosis of non-classical congenital adrenal hyperplasia. She was treated with estradiol, glucocorticoids, and transdermal testosterone. After hormonal levels were lowered, *in vitro* fertilization cycles were initiated, and the patient had a spontaneous ovulation. In case II, a 20-year-old Caucasian Israeli-Arab female presented for infertility evaluation owing to her oligomenorrhea. Her vitals and physical examination had normal results. The investigation of her abnormal hormonal profile led her to be referred to genetic testing, where the results showed the same genetic mutation as seen in case I.

**Conclusion:**

Both cases highlight the distinctiveness of the condition, where an identical mutation in the gene responsible for the same enzyme can bring about diverse phenotypes. Case I offers a potential treatment protocol for this rare disorder.

## Introduction

Congenital adrenal hyperplasia (CAH) is a group of autosomal recessive disorders that are caused by deficiencies of enzymes in steroidogenesis, leading to abnormal levels of glucocorticoids, mineralocorticoids, and sex steroids. 17-α-Hydroxylase deficiency (17-OHD) is the rarest form of CAH, comprising < 1% of all cases. 17-OHD is caused by a mutation in the *CYP17A1* gene and may lead, depending on the severity, to impaired synthesis of estrogens, androgens, and cortisol in the adrenal glands and gonads. Patients usually exhibit abnormal levels of sex hormones and may also show abnormally high levels of mineralocorticoids. Classic symptoms of 17-OHD may include hypertension, hypokalemia, and hypogonadism [1]. A partial deficiency of the enzyme, similarly to *CYP21A1* mutation, would lead to a milder phenotype that typically would be diagnosed in puberty, and often presents in female patients with amenorrhea and anovulation. The syndrome may also include sexual infantilism, impaired secondary sexual development, and primary infertility and is classified as non-classical congenital adrenal hyperplasia (NCCAH). The enzyme encoded by the *CYP17A1* gene has two sequential roles; its hydroxylase action promotes the conversion of pregnenolone and progesterone to 17 OH-pregnenolone and 17 OH-progesterone, respectively. The second action involves its lyase-desmolase action that converts 17-OH pregnenolone to dehydroepiandrosterone (DHEA) and 17 OH-progesterone to androstenedione. The resulting hormone deficiencies depend on which of the enzymes two actions is compromised, as the bottle neck created by the relative dysfunction of the enzymatic step may result in a relative lack of its hormonal products and accumulation of their precursors. These hormonal aberrations in turn govern the phenotype. The condition has no consensus treatment and presents considerable challenges to clinicians [2]. We present two cases of patients, that despite being homozygote for the same *CYP17A1* mutation, presented with dissimilar phenotype.

## Case I

M.S. a Caucasian Israeli-Arab presented to our clinic at age 24 years after 6 years of primary infertility. Prior to starting combined oral contraceptives (COC), she had three laparoscopic ovarian cystectomies (2016–2017), secondary to recurrent simple cyst formation. Before her first visit to our clinic she had one cycle of *in vitro* fertilization (IVF) treatment, retrieving six oocytes and producing three embryos, which were transferred but no pregnancy was achieved. In 2019, while still under COC, her hormonal profile was tested. Results showed an abnormally low level of E2 (40 pmol/L), and high levels of both progesterone (13.1 nmol/L, repeat progesterone levels were 16 and 29 nmol/L) and follicle-stimulating hormone (FSH; 19.6 IU).

**Fig. 1:**
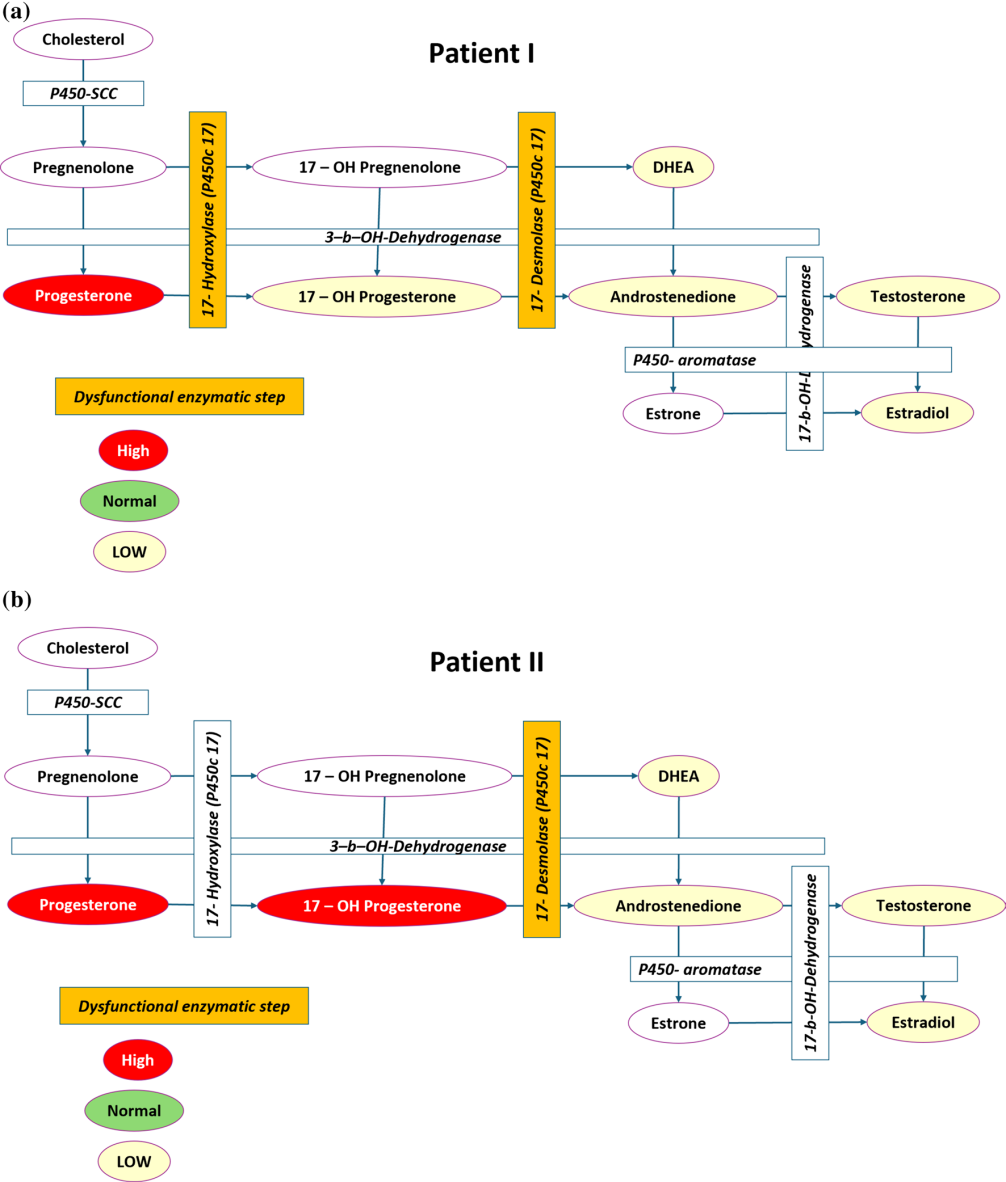
Steroidogenesis enzymatic pathway. The figure demonstrates the enzymatic steps in squares and the steroid products in ellipses. Abnormally increased serum concentration of a certain steroid is marked in red, normal in green, and low in yellow. The dysfunctional enzymatic step is colored in orange. Uncolored steroids represent steroids for which serum concentration was not measured. **A** shows the steroid measurements for the patient described in case I and the deduced dysfunctional enzymatic steps while **B** described the steroid concentration and dysfunctional enzymatic step for case II

Her physical examination was negative for hirsutism, acne, and hypertension. Further evaluation revealed very low levels of anti-mullerian hormone (AMH; 0.22 ng/mL), a normal female karyotype and *FMR1* CGG repeat number. Vital signs were normal. Following several consecutive elevated serum progesterone measurements, with low to normal 17 OH-progesterone, a post COC cessation random complete endocrine profile was sent, which was also remarkable for low DHEA, low androstenedione, and extremely low serum concentration of estradiol and testosterone (Fig. 1A). She was then referred to genetic counseling for steroidogenic enzyme gene mutations. Results showed a biallelic mutation in the *CYP17A1* gene (c.1486 > T(hom), P.Arg496Cys), affecting the coding to 17-hydroxylase-desmolase enzymes. Her husband, a first-degree cousin, was diagnosed as a carrier of the same mutation and therefore pregestational testing for gene mutations (PGTM) was advised. COC treatment was stopped and replaced with medical treatment that was designed to lower the serum concentration of progesterone and FSH using a combination of estradiol (Estrofem 2 mg OD, Novo Nordisk, Bagsvaered, Denmark) and glucocorticoids (dexamethasone 0.5 mg OD, Rekah, Holon, Israel). She was also prescribed with transdermal testosterone (Testomax 12.5 mg per day, Actavis, Dublin, Ireland) to replace the deficiency and improve ovarian response. Table 1 describes the changes in serum estradiol, progesterone (P4), FSH, luteinizing hormone (LH), testosterone, as well as ultrasound measurements of follicle size and endometrial thickness. As detailed in the table, following 2 weeks of treatment her serum progesterone and FSH had normalized, and a spontaneous ovulation was recorded 23 days from treatment initiation. The patient underwent two IVF cycles; first cycle using antagonist protocol combined with the current priming regimen that resulted in two extracted oocytes, one fertilized then frozen. The second cycle employed a similar protocol and produced four extracted oocytes of which three were fertilized. Since the endometrium was exposed to chronically high serum progesterone, the patient presented with persistently thin endometrium. We therefore selected to vitrify all embryos until a proper endometrial thickness would be attained. Table 2 describes the egg retrieval, embryo transfer and PGTM cycles detailing the number of retrieved oocytes, embryos created, transfered and frozen as well as the outcome of the treatment cycle.

**Table 1:** Case I serum estradiol (E2), progesterone, (P4), FSH, LH, testosterone as well as follicle size and endometrial thickness

Day of treatment	1	10	17	23	31	37	46	51	58	65	79	93	100
E2, pmol/L	0	160	441	389	276	325	221	262	162	250	268	147	188
P4, nmol/L	29	12.7	5.7	15.1	0.63	0.49	0.69	0	1.17	0.73	3.44	3.58	2.31
FSH, IU	19.6	–	4.97	7.35	5.2	5.22	5.56	5.72	6.36	6.77	4.9	7.49	7.57
LH, IU	–	–	–	6.5	3.1	1.74	1.98	1.56	1.17	2.87	1.85	1.85	4.1
Test., nmol/L	0	0	0	0	–	25.27	36.17	28	3.13	0.73	24.71	24.71	0
Follicle size in mm													
Rt	–	S	15,15,11	CL14	S	S	9,9, + +	S	S	S	S	S	11 + +
Lt	–	S	S	S	S	S	S	S	S	S	S	CL17	S
Endometrial thickness (mm)	–	3	4.6	3.6	3	3	2.6	2.9	2.2	2.2	2.2	3.9	2.8

**Table 2:** Description of IVF treatments for patient I with details on the number of retrieved oocytes, total embryos, embryos transferred and cryopreserved

Months since first IVF		Oocytes	Embryos	Transferred	Frozen	Pregnancy
0	ICSI	2	1	0	1	–
2	ICSI	4	3	0	2	–
3	ICSI	3	3	0	3	–
4	PGTM—5 good for biopsy, 3 affected, 2 carriers					
4	FET	–	–	1	–	–
8	FET	–	–	1	–	P
10	ICSI	7	5	0	5	–
11	ICSI	14	6	0	6	–
12	PGTM—9 good for biopsy, 2 affected, 3 carriers, 1 no result					
12	FET	–	–	1	–	–
15	FET	–	–	1	–	–
18	FET	–	–	1	–	–
20	ICSI	8	2	0	2	–
21	ICSI	6	1	0	2	–
23	ICSI	7	4	0	4	–
25	PGTM—7 good for biopsy, 2 affected, 4 carriers, 1 no result					
25	FET	–	–	2	–	P
28	FET	–	–	1	–	–
29	FET	–	–	1	–	P

The table also details the PGTM details with the number of biopsied embryos, found to be carriers, affected and those with no result

The patient had then started ovarian stimulation for IVF and PGTM. Owing to the very low ovarian reserve, she was assigned to the antagonist protocol with transdermal testosterone priming, as well as treatment with dexamethasone and low dose oral estradiol (Estrofem 1 mg) to maintain low serum progesterone and FSH. Ovarian stimulation was achieved using 300 IU of Pergoveris daily (Merck, Darmstadt, Germany). Since the number of embryos was less than optimal to proceed with PGTM, embryos were banked in the first two IVF cycles. Biopsy and analysis for the presence of the *CYP17A1* was conducted only after five embryos were available to biopsy at the end of the third cycle. Results of the PGTM showed two transferable embryos, that were transferred in two separate frozen embryo transfers (FETs). Endometrial preparation was done using dexamethasone and high dose oral and vaginal estradiol. The chronic exposure of the lining to progesterone led to endometrial resistance, which was overcome only after a month of endometrial stimulation with high dose estradiol and very low serum progesterone. The second FET resulted in a clinical pregnancy that unfortunately was miscarried at 7 weeks. A workup post the miscarriage revealed that the patient is also a carrier of the Factor V Leyden mutation, and subcutaneous Enoxaparin at 40 mg (Clexane, Sanofi, Paris, France) was added to her treatment protocols henceforth.

The patient had five more cycles of ovarian stimulation that showed increasingly higher numbers of retrieved oocytes and two more PGTMs. She had one more pregnancy that unfortunately was miscarried as well. The patient is currently in the second trimester of pregnancy following the last transfer. During her treatment, she had also been diagnosed with non insulin dependent diabetes that was secondary to weight gain and likely exacerbated by chronic exposure to glucocorticoids, and responded well to treatment with metformin.

## Case II

The second patient H.S., a Caucasian Israeli-Arab, G0P0 20-year-old who presented with oligomenorrhea for infertility evaluation. Her blood pressure was normal as well as the rest of her physical examination. Ultrasound examination was remarkable for a simple ovarian cyst. A post negative beta-human chorionic gonadotropin (BHCG) test failure to respond to progesterone withdrawal prompted a random endocrine profile. The profile revealed an abnormally low level of E2 (< 70 pmol/L) and a high P4 level (25.6 nmol/L). Other serum hormone measurements included FSH (8.3 IU), LH (10 IU), prolactin (PRL; 426 mU/L), and testosterone (< 0.45 nmol/L). In the following month, her progesterone, estradiol, and FSH levels were tracked. Table 3 includes these results, that showed constantly elevated P4 (from 19.4 nmol/L to 39.93 nmol/L in 6 weeks) and FSH levels. E2 levels were still lower than normal. Her 17 OH-P level was also abnormally high (24 nmol/L). The constantly elevated progesterone, high 17-OH-progesterone, and low serum levels of androstenedione, testosterone, and estradiol suggested that the bottle neck centered around the 17-hydroxylase enzyme (Fig. 1B). The patient was referred for genetic testing for a mutation in the *CYP17A1* gene. The result showed an identical mutation c.1486 > T(hom), P.Arg496Cys in the *CYP17A1* seen in case I. Her partner was tested and was found not to carry this mutation.

**Table 3:** Case II serum estradiol (E2), FSH, and progesterone (P4)

	17/11/2022	20/11/2022	20/12/2022	29/12/2022
Estradiol, pmol/L	117	103	–	109
FSH, IU	14.37	12.46	–	10.6
Progesterone, nmol/L	19.4	20.5	25.03	39.93

## Discussion

**Fig. 2:**
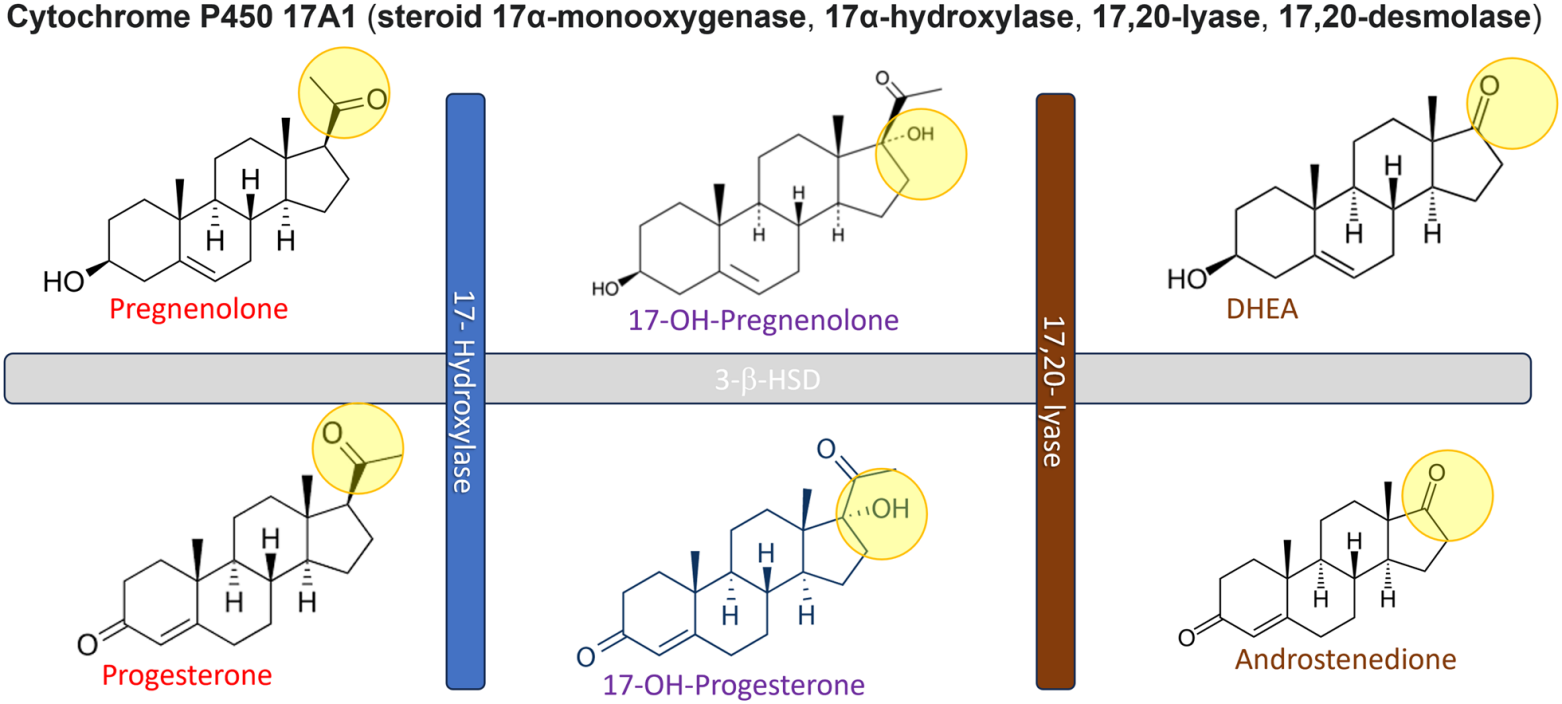
The two actions of the 17-hydroxylase-17,20-lyase enzyme, in the first step it adds a hydroxyl group to carbon 17 on pregnenolone and progesterone to create 17-hydroxypregnenolone and 17-hydroxyprogesterone, respectively. In the second step it splits the sidechain off the steroid nucleus to create dehydroepiandrosterone and androstenedione, respectively

Non classical CAH caused by the mutation in *CYP17A1* corresponding to 17-OHD has an estimated incidence of 1 in 50,000–100,000 individuals [3]. The resulting hormonal deficiency and associated phenotype is dependent on the dysfunctional enzymatic activity. The enzymes that promote the formation of steroids from the cholesterol building block are ubiquitous and can be found in the cells of the adrenal cortex and gonads (Fig. 1). The enzyme encoded by the *CYP17A1* is unique, as it promotes two consecutive steps in the steroidogenesis pathway; the hydroxylation of pregnenolone and progesterone on carbon 17 to their corresponding 17-OH products, and the lyase activity to split the sidechain off the steroid nucleus (Fig. 2). As seen in Fig. 1, the bottle neck created by this enzyme deficiency is crucial to understanding the phenotype.

M.S. had a phenotype that was consistent with a deficiency in both the 17α-hydroxylase enzyme and 17,20 lyase enzyme. P4 is a precursor of 17-OH-progesterone (17α-OH is responsible for the conversion, Fig. 1A), the patients’ abnormally high levels of P4 and low to normal 17-OH-progesterone are indicative of this enzymatic deficiency. Furthermore, the extremely low levels of estradiol, testosterone, DHEA, and androstenedione suggest the lack of 17,20 Lyase enzyme, which is a key part in the making of both androgens and estrogens (Fig. 1A). The phenotype she displayed can be understood through her abnormal hormonal levels; her low levels of E2, removing the negative feedback from pituitary FSH secretion led to elevated FSH, and the resulting growth of follicles but not ovulation, while the chronically elevated serum progesterone prevented the LH surge and ovulation, thus causing the formation of her recurring ovarian cysts. The multiple surgeries the patient in case I had to remove the ovarian cysts are the likely culprit in the loss of ovarian reserve, as seen by her abnormally low AMH level and poor response. The poor ovarian response might have been further exacerbated by the complete lack of androgens, that would otherwise increase the expression of FSH receptors on follicular granulosa cells [4]. Unlike cases of severe 17-OHD that may lead to cortisol deficiency and the adrenocorticotropic hormone (ACTH) induced compensation that may lead to prebottleneck hyperaldosteronism and the associated hypertension and hypokalemia, both patients presented had a mild 17-OHD, and had no clinical cortisol deficiency or hyperaldosteronism. The compensation for the relative lack of cortisol in both patients led to chronically elevated serum progesterone that responded well to glucocorticoid replacement therapy. However, interestingly, despite having an identical mutation in the CYP17A1 gene, the serum concentration of 17-OH- progesterone in the two patients was different. In case I serum 17-OH-progesterone was low-normal while in patient II it was significantly elevated (Fig. 1). This could be explained by a proportional dysfunction in both actions of the enzyme in patient I, while in patient II the 17,20 lyase action was more compromised.

In patient I, the treatment was complicated by the presence of diminished reserve, need for PGTM, and other comorbidities that led to the relatively poor outcomes. The decision to test the embryos with PGTM was guided by the perceived risk of a more severe phenotype in the offspring and ambiguous genitalia in a male fetus. The treatment protocol M.S. underwent was effective on three levels; dexamethasone suppressed the overproduction of progesterone, estradiol supplementation lowered serum FSH and ovarian cyst formation, and transdermal testosterone gradually improved ovarian response and estradiol production. Treatment proved effective, as M.S. had a spontaneous ovulation 23 days into the treatment.

## Conclusion

The two presented cases exemplify the challenges posed by this rare clinical condition in attempting to achieve a pregnancy. The presentation also highlights the uniqueness of the condition in which an identical mutation in the gene encoding for the same enzyme may lead to a different phenotype.

## Data Availability

Data available per request.
